# Accuracy of 12 Wearable Devices for Estimating Physical Activity Energy Expenditure Using a Metabolic Chamber and the Doubly Labeled Water Method: Validation Study

**DOI:** 10.2196/13938

**Published:** 2019-08-02

**Authors:** Haruka Murakami, Ryoko Kawakami, Satoshi Nakae, Yosuke Yamada, Yoshio Nakata, Kazunori Ohkawara, Hiroyuki Sasai, Kazuko Ishikawa-Takata, Shigeho Tanaka, Motohiko Miyachi

**Affiliations:** 1 Department of Physical Activity Research National Institutes of Biomedical Innovation, Health and Nutrition Tokyo Japan; 2 Faculty of Sport Sciences Waseda University Tokorozawa Japan; 3 Department of Nutrition and Metabolism National Institutes of Biomedical Innovation, Health and Nutrition Tokyo Japan; 4 Graduate School of Engineering Science Osaka University Osaka Japan; 5 Section of Healthy Longevity Research National Institutes of Biomedical Innovation, Health and Nutrition Tokyo Japan; 6 Faculty of Health and Sport Sciences University of Tsukuba Ibaraki Japan; 7 Graduate School of Informatics and Engineering University of Electro-Communication Tokyo Japan; 8 Graduate School of Arts and Sciences The University of Tokyo Tokyo Japan

**Keywords:** physical activity, accelerometry, energy expenditure, indirect calorimetry, doubly labeled water

## Abstract

**Background:**

Self-monitoring using certain types of pedometers and accelerometers has been reported to be effective for promoting and maintaining physical activity (PA). However, the validity of estimating the level of PA or PA energy expenditure (PAEE) for general consumers using wearable devices has not been sufficiently established.

**Objective:**

We examined the validity of 12 wearable devices for determining PAEE during 1 standardized day in a metabolic chamber and 15 free-living days using the doubly labeled water (DLW) method.

**Methods:**

A total of 19 healthy adults aged 21 to 50 years (9 men and 10 women) participated in this study. They followed a standardized PA protocol in a metabolic chamber for an entire day while simultaneously wearing 12 wearable devices: 5 devices on the waist, 5 on the wrist, and 2 placed in the pocket. In addition, they spent their daily lives wearing 12 wearable devices under free-living conditions while being subjected to the DLW method for 15 days. The PAEE criterion was calculated by subtracting the basal metabolic rate measured by the metabolic chamber and 0.1×total energy expenditure (TEE) from TEE. The TEE was obtained by the metabolic chamber and DLW methods. The PAEE values of wearable devices were also extracted or calculated from each mobile phone app or website. The Dunnett test and Pearson and Spearman correlation coefficients were used to examine the variables estimated by wearable devices.

**Results:**

On the standardized day, the PAEE estimated using the metabolic chamber (PAEEcha) was 528.8±149.4 kcal/day. The PAEEs of all devices except the TANITA AM-160 (513.8±135.0 kcal/day; *P*>.05), SUZUKEN Lifecorder EX (519.3±89.3 kcal/day; *P*>.05), and Panasonic Actimarker (545.9±141.7 kcal/day; *P*>.05) were significantly different from the PAEEcha. None of the devices was correlated with PAEEcha according to both Pearson (r=−.13 to .37) and Spearman (ρ=−.25 to .46) correlation tests. During the 15 free-living days, the PAEE estimated by DLW (PAEEdlw) was 728.0±162.7 kcal/day. PAEE values of all devices except the Omron Active style Pro (716.2±159.0 kcal/day; *P*>.05) and Omron CaloriScan (707.5±172.7 kcal/day; *P*>.05) were significantly underestimated. Only 2 devices, the Omron Active style Pro (r=.46; *P*=.045) and Panasonic Actimarker (r=.48; *P*=.04), had significant positive correlations with PAEEdlw according to Pearson tests. In addition, 3 devices, the TANITA AM-160 (ρ=.50; *P*=.03), Omron CaloriScan (ρ=.48; *P*=.04), and Omron Active style Pro (ρ=.48; *P*=.04), could be ranked in PAEEdlw.

**Conclusions:**

Most wearable devices do not provide comparable PAEE estimates when using gold standard methods during 1 standardized day or 15 free-living days. Continuous development and evaluations of these wearable devices are needed for better estimations of PAEE.

## Introduction

### Background

Physical activity (PA) has been reported to reduce the incidence of and mortality because of several noncommunicable diseases, including cardiovascular disease, stroke, and some types of cancer [1-3]. To promote or maintain PA, self-monitoring using pedometers and accelerometers has been considered effective [4]. However, the validity of estimating the amount of PA or PA energy expenditure (PAEE) detected using wearable devices has not been sufficiently established. Previously, we simultaneously examined the validity of total energy expenditure (TEE) estimated by 12 wearable devices during 1 standardized day in a metabolic chamber and 15 free-living days using the doubly labeled water (DLW) method [5]. This study allowed the ranking of daily individual TEE (ρ=.80-.88), but absolute values varied widely among devices and differed significantly from the criterion under free living. Moreover, it is better to estimate accurately not only TEE but also daily PAEE because TEE is mainly determined by the basal metabolic rate (BMR) rather than PA [6].

Several studies have tested the validity of wearable devices for estimating energy expenditure (EE) during some activities [7-14]. However, most have compared EE estimated by wearable devices and standard reference measures estimated by an expired gas analysis during very short structured activities in laboratories [7-9,11-13]. EE measured during such study designs also included resting EE (REE) or BMR, which do not reflect net PAEE. The BMR accounts for a substantial proportion of TEE and is relatively constant from day to day. In contrast, PAEE contributes to TEE to a lesser extent, but it is a fairly variable component that allows the opportunity to increase TEE [6]. Due to the relationship between the amount of PA and health outcomes, accurate estimations of the net PAEE using wearable devices are required, especially under free-living conditions that use wearable devices. Various wearable devices are available for consumer purchase [15], but little is known about their validity.

### Objectives

In this study, we evaluated the validity of consumer-based and research-grade wearable devices for estimating PAEE values without the BMR or REE. We developed 2 designs: (1) standardized day for PAEE estimated using a metabolic chamber and (2) 15 free-living days for PAEE estimated using the DLW method.

## Methods

### Participants

A total of 21 healthy adults aged 21 to 50 years (9 men and 12 women) participated in this study. None of the participants had chronic diseases that could affect their metabolism or daily PA. Their body mass index (BMI) values were within the normal range (18.5-25.0 kg/m^2^). Of 21 participants, 2 were excluded from all analyses: 1 because personal information in the JAWBONE UP24 (Jawbone, San Francisco, CA, USA) app during the 15 free-living days experiment had been set incorrectly, and the other because data from the metabolic chamber during the 1 standardized day experiment was incorrect because of instrument failure. Finally, 19 participants (9 men and 10 women) were included in this analysis. All procedures were reviewed and approved by the Ethics Review Board of the National Institute of Health and Nutrition (kenei-4-02). All participants provided written informed consent.

### Wearable Devices

The consumer-based wearable devices used in this study were selected based on the following criteria: they were the most popular devices in Japan according to several marketing websites based on their sales ranking (eg, Amazon, Japan website[16] or kakaku website[17] as of December 1, 2014); the app could be displayed in Japanese on a mobile phone or website; and the clock settings of the app or device could be manipulated. We needed to change the clock setting from 9:00 am to 9:00 am the next day to 12:00 am to 12:00 am the next day to obtain the TEE for an entire day when participants used the metabolic chamber. A total of 8 wearable devices, including the Fitbit Flex (Fitbit, San Francisco, CA, USA), JAWBONE UP24, Misfit Shine (Misfit Wearables, Burlingame, CA, USA), EPSON PULSENSE (SEIKO EPSON, Nagano, Japan), Garmin Vivofit (Garmin, Olathe, KS, USA), TANITA AM-160 (TANITA, Tokyo, Japan), Omron CaloriScan HJA-401F (OMRON HEALTHCARE, Kyoto, Japan), and Withings Pulse O2 (Withings, Issy-les-Moulineaux, France), were selected for this study (Table 1). In addition, 4 research-grade wearable devices, namely, Omron Active style Pro (OMRON HEALTHCARE, Kyoto, Japan), Panasonic Actimarker EW4800 (Panasonic, Osaka, Japan), SUZUKEN Lifecorder EX (SUZUKEN, Aichi, Japan), and ActiGraph GT3X (ActiGraph, Pensacola, FL, USA), were used in this study (Table 1). All devices had a built-in accelerometer. Of 12 wearable devices, 5 (Fitbit Flex, JAWBONE UP24, Misfit Shine, EPSON PULSENSE, and Garmin Vivofit) were placed on the nondominant wrist, 2 (TANITA AM-160 and Omron CaloriScan) were placed in a pocket, and 3 (Withings Pulse O2, Omron Active style Pro, Panasonic Actimarker, SUZUKEN Lifecorder EX, and ActiGraph GT3X) were placed on the waist. The position on the wrist or waist was randomly chosen for each participant, and each participant placed the devices in the same position throughout the experiments.

**Table 1:** Wearable devices used in the present study, basal metabolic rates extracted from each device, and information about invalid days and non-wearing time in 15 free-living days.

Number	Devices	Placement	Basal metabolic rates^a^ (kcal/day), average (SD)	15 free-living days		
				Invalid days^b^	Nonwearing time in valid day	
					min/day, average (SD)	kcal/day^c^, average (SD)
1	Fitbit Flex	wrist	1360.4 (195.2)	1	42.4 (18.4)	26.9 (23.4)
2	JAWBONE UP24	wrist	1312.6 (157.1)	0	40.1 (13.0)	25.4 (22.9)
3	Misfit Shine^d^	wrist	1708.0 (245.9)	15	40.4 (13.2)	26.1 (23.1)
4	EPSON PULSENSE^d^	wrist	1616.8 (179.8)	4	42.2 (13.5)	26.4 (22.3)
5	Garmin vivofit^d^	wrist	1630.2 (234.8)	0	39.4 (12.9)	25.2 (23.0)
6	TANITA AM-160^d^	pocket	1410.4 (211.5)	1	42.6 (14.3)	29.3 (29.0)
7	Omron CaloriScan^d^	pocket	1291.7 (186.2)	1	42.6 (14.3)	29.3 (29.0)
8	Withings Pulse O2^d^	waist	1608.9 (228.4)	1	45.5 (13.2)	33.5 (30.8)
9	Omron Active style Pro^d^	waist	1304.5 (188.5)	0	43.1 (13.8)	30.6 (31.3)
10	Panasonic Actimarker	waist	1327.5 (172.4)	0	43.1 (13.8)	30.6 (31.3)
11	SUZUKEN Lifecorder EX	waist	1327.4 (171.9)	0	43.1 (13.8)	30.6 (31.3)
12	ActiGraph GT3X^e^	waist	—^f^	2	42.9 (14.2)	30.5 (31.4)

^a^Basal metabolic rates were extracted from each app.

^b^Total invalid days in 19 participants during 15 days.

^c^The energy expenditure (kcal) in non-wearing time on a valid day was calculated based on time and METs reffered to the Compendium of Physical Activities.

^d^*P*<.05 vs BMR in metabolic chamber (1355.0±234.9 kcal/day).

^e^ActiGraph indicates only PAEE on its application.

^f^Not applicable.

### Experimental Design

A total of 2 experiments were conducted to test the validity of the wearable devices: 1 used the metabolic chamber method during 1 standardized day, and the other used the DLW method during 15 free-living days. These 2 methods were used as the standard to determine TEE [18,19]. For the 1-day standardized experiment, participants visited the laboratory 2 hours before the start of the experiment (7:00 am) after an overnight fast of at least 10 hours. Then, height, weight, and body composition were measured. After setting and wearing 12 wearable devices, participants entered the metabolic chamber before 9:00 am and completed 24-hour metabolic chamber measurements (9:00 am to 9:00 am the next day) using a standardized protocol that included various activities common during daily life such as eating 3 meals, watching television (TV), using a computer, cleaning, and walking on a treadmill (Table 2). Each participant’s energy intake for meal was calculated by multiplying each BMR by 1.6, which was the PA level (PAL) assumed for a standardized day. The meal was served 3 times per day, and the total energy intake was equally divided into 3 times. The participants were instructed to eat all the meals that were served, and they were not allowed to eat any other foods in the metabolic chamber. However, they were permitted to drink water freely. The average metabolic equivalents (METs) estimated using the compendium of physical activities [20] and previous studies [21-24] for this protocol was 1.37 METs, and the mean PAEE estimated using the estimated METs×hour and participants’ weight was 447.0±66.8 kcal/day. Participants wore all the wearable devices during their waking hours without removing them. The 5 devices on the wrist were worn even while sleeping.

**Table 2:** Timetable for metabolic chamber on a standardized day.

Time	Activity
8:45	Entry in the room
09:00 – 09:30	TV watching
09:30 – 10:30	Breakfast; rice, chicken soup, macaroni salad, and sausage
10:30 – 11:00	Computer work
11:00 – 11:30	Reading a book on a stand
11:30 – 12:00	Folding the laundry
12:00 – 12:30	Cleaning
12:30 – 12:30	Walking (4.0 km/h), including 5 min of rest after walking
13:00 – 13:30	Walking (5.6 km/h), including 5 min of rest after walking
13:30 – 14:00	TV watching
14:00 – 15:00	Lunch; stir-fried vegetables & seafood on rice, cooked beans, egg, and miso soup
15:00 – 15:30	Computer work
15:30 – 16:00	TV watching
16:00 – 16:30	Desk work
16:30 – 17:00	Cleaning
17:00 – 17:30	Walking (4.0 km/h), including 5 min of rest after walking
17:30 – 18:00	Walking (5.6 km/h), including 5 min of rest after walking
18:00 – 18:30	TV watching
18:30 – 19:30	Dinner; rice, hamburg steak, salad, ham, and, vegetable soup
19:30 – 20:00	Computer work
20:00 – 20:30	Reading a book on a stand
20:30 – 21:00	Desk work
21:00 – 21:30	Computer work
21:30 – 22:00	TV watching
22:00 – 22:30	Folding the laundry
22:30 – 23:00	Readying oneself for sleep
23:00 – 07:00	sleep
07:00 – 07:15	lying
07:15 – 08:00	Supine posture
08:00 – 09:00	TV watching
9:10	Exit from the room

During the experiment involving 15 free-living days, participants visited the laboratory in the morning after an overnight fast of at least 10 hours and underwent measurements of height, weight, and body composition. After collecting baseline urine samples, DLW dosing was performed in the laboratory. A premixed dose containing approximately 0.06 g/kg of body weight of ^2^H_2_O (99.8 atom%; Cambridge Isotope Laboratories, MA, USA) and 1.4 g/kg of body weight of H_2_^18^O (10.0 atom%; Taiyo Nippon Sanso, Tokyo, Japan) was administered orally to each participant. All participants collected their urine samples in air-tight parafilm-wrapped containers at the same time on days 1, 2, 3, 8, 9, 13, 14, and 15 after the baseline day (day 0) during free-living conditions.

Participants wore all the wearable devices when they were awake, but they did not wear them during water-related physical activities, physical activities during which the devices were difficult to wear, or when the battery was charging. Of 12 wearable devices, 5 were worn on the wrist even while sleeping. After 15 free-living days, all urine samples were collected and stored at −30ºC until they were analyzed. Dietary assessments using a brief self-administered diet history questionnaire [25] were conducted to calculate the food quotient (FQ) after 15 days. Logs for time awake, time asleep, nonwearing time, and PA during nonwearing time were completed for 15 days by each participant. PAEE during the nonwearing time was calculated based on the recorded time and METs that were referred to the *Compendium of Physical Activity* [20].

### Data Reduction for Each Wearable Device

For the experiment involving 15 free-living days, the days were considered valid when participants wore the wearable devices for more than 10 hours/day [26]. However, we included 1 day when a participant slept for more than 14 hours and, therefore, did not wear the devices for more than 10 hours. The minimum number of valid days was defined as 10 days, and all participants fulfilled this requirement. The mean PAEE of valid days was used for the experiment involving 15 free-living days.

The PAEE for each device (PAEE_dev_) was calculated by subtracting the BMR and 0.1×TEE as diet-induced thermogenesis (DIT) from TEE estimated by each device (TEE_dev_). The PAL for each device (PAL_dev_) was calculated by dividing the TEE by the BMR. The BMR for each device (BMR_dev_) was calculated using the app. The SUZUKEN Lifecorder EX did not show the BMR_dev_ on the app, but the computation method for the BMR using the body surface area and coefficient of the BMR was provided in its instructions; therefore, we calculated the BMR according to those instructions. Because some devices did not show the individual predicted BMR in the device app, including the Fitbit Flex, Misfit Shine, Omron CaloriScan, and Withings Pulse O2, the TEE values of a day when the devices were stationary for the entire day were used as the BMR_dev_. However, the Omron CaloriScan provided information, indicating that the DIT is included in the TEE when it was stationary for the entire day. Therefore, we did not subtract the DIT when PAEE was calculated using TEE. The ActiGraph GT3X showed only PAEE, not TEE; therefore, we used only the PAEE shown by the ActiGraph GT3X software.

### Anthropometry and Body Composition

Height and body weight were measured on both experiment days, and each profile was used for each experiment. BMI (kg/m^2^) was calculated, and body composition was determined using a bioelectrical impedance analysis (Inner Scan BC-600; TANITA).

### Measurement of Energy Expenditure on a Standardized Day Using the Metabolic Chamber

An open-circuit, indirect metabolic chamber equipped with a bed, desk, chair, TV, toilet, sink, and treadmill was used to measure EE. The temperature and relative humidity in the room were controlled at 25ºC and 55%, respectively. Oxygen and carbon dioxide concentrations of the air supply and exhaust were measured using mass spectrometry (ARCO-1000A-CH; Arco System, Kashiwa, Japan). The flow rates of the exhausts from the chamber were measured using pneumotachography (FLB1; Arco System). Oxygen uptake (VO_2_) and carbon dioxide output (VCO_2_) were determined based on the concentrations of the inlet and outlet air flows from the chamber and the flow rate of the exhausts from the chamber, respectively. TEE from 9:00 am the first day until 9:00 am the next day was estimated from VO_2_ and VCO_2_ using Weir equation (TEE_cha_). The BMR was measured in the supine position for 45 min during the morning (BMR_cha_). The PAEE during 1 standardized day (PAEE_cha_) was calculated by subtracting the BMR_cha_ and 0.1×TEE_cha_ from TEE_cha_. The PAL during 1 standardized day (PAL_cha_) was calculated by dividing the TEE_cha_ by the BMR_cha_.

### Measurement of Energy Expenditure During 15 Free-Living Days Using the Doubly Labeled Water Method

Gas samples for the isotope ratio mass spectrometer (IRMS) were prepared by maintaining the equilibration of the urine sample with gas. CO_2_ was used to equilibrate^18^O, and H_2_ was used to equilibrate^2^H. The platinum (Pt) catalyst was used for equilibration of^2^H. Gas samples for CO_2_ and H_2_ measurements were analyzed using IRMS (Sercon 20-20; Sercon Ltd, Crewe, UK). Each sample and its corresponding reference were analyzed in triplicate. The^2^H and^18^O zero-time intercepts and elimination rates (kd and ko) were calculated using the least-squares linear regression method on the natural logarithm of the isotope concentration as a function of the elapsed time from dose administration. Zero-time intercepts were used to determine the isotope pool sizes. A quality check was conducted according to the International Atomic Energy Agency book [27]. The memory effects of the IRMS were eliminated and checked using additional samples when the expected isotope ratio difference was high (eg, days 2-8), and the potential drift of the IRMS was corrected mathematically using standardized working criteria and checked for accuracy and precision using another working criterion at regular intervals in a series of measurements and between different measurement days. The samples obtained from 1 participant were analyzed in 1 series of measurements in 1 day to minimize the effects of day-to-day variation. The dilution space ratio of^2^H (Nd) and^18^O (No) of all 21 participants was 1.036±0.010 (range 1.021-1.056), which was an acceptable value according to a previous review of a large database [28]. Therefore, total body water (TBW) was calculated from the mean value or the isotope pool size of^2^H divided by 1.041 and that of^18^O divided by 1.007. The carbon dioxide production rate (rCO_2_) was calculated as follows: rCO_2_=0.4554×TBW×(1.007 ko−1.041 kd), for which we assumed that isotope fractionation applies only to breath water using equation A6 by Schoeller et al [29] with the revised dilution space constant provided by Racette et al [30]. The TEE (TEE_dlw_) was calculated using a modified Weir formula based on the rCO_2_ and FQ [31] as follows: TEE (kcal/day)=1.1 rCO_2_+3.9 rCO_2_/FQ.

The PAEE during free-living days (PAEE_dlw_) was calculated by subtracting the BMR_cha_ and 0.1×TEE_dlw_ from TEE_dlw_. The PAL during free-living days (PAL_dlw_) was calculated by dividing TEE_dlw_ by BMR_cha_.

### Statistical Analysis

Data were expressed as mean (standard deviation). The Dunnett test, for which standard criteria were set as references, was used for comparing variables estimated by wearable devices during the use of the metabolic chamber method and the DLW method. The mean absolute percent errors (MAPEs) relative to the PAEE values estimated using standard methods were calculated to provide an indicator of the overall measurement error. The Pearson and Spearman correlation coefficients were used to examine the relationship between standard criteria and variables estimated by wearable devices. Modified Bland-Altman plots [32] were used to test proportional biases between standard methods and devices, and the correlation coefficient of the standard criteria and the differences between the standard criteria and each device were examined for significance. During all analyses, *P*<.05 was considered statistically significant. All statistical analyses were performed with SPSS version 20.0 for Windows (IBM SPSS Japan Inc, Tokyo, Japan).

## Results

### Descriptive Results

Participants were aged 32.3±9.6 years. Their BMI and percentage body fat ranged from 18.5 to 24.8 kg/m^2^ and from 14.8% to 32.2%, respectively. Although there was no invalid day during the standardized 1-day experiment, 25 invalid days were identified during the 15-day free-living experiment (Table 1), which corresponded to 8.8% of all experiment days (19 participants×15 days). Invalid days often occurred with the Misfit Shine because a few of these devices became loose without the knowledge of the participant and with EPSON PULSENSE because the battery quickly died. The average nonwearing time except for sleeping for each device ranged from 39.4±12.9 to 45.5±13.2 min/day, which corresponded to 25.2±23.0 to 33.5±30.8 kcal/day (Table 1). The most frequent activities during nonwearing time were bathing and showering (289 cases/19 participants×15days). There were 62 other activities including TV watching, deskwork, dressing, and exercise. The corresponding time and intensity for these activities were 5 to 450 min and 1.3 to 6.3 METs, respectively. The BMR_cha_ was 1355.0±234.9 kcal/day. Several devices showed higher BMR_dev_ than BMR_cha_ (*P*<.05), including the Misfit Shine, EPSON PULSENSE, Garmin Vivofit, TANITA AM-160, and Withings Pulse O2 (Table 1).

### Metabolic Chamber Study

**Figure 1:**
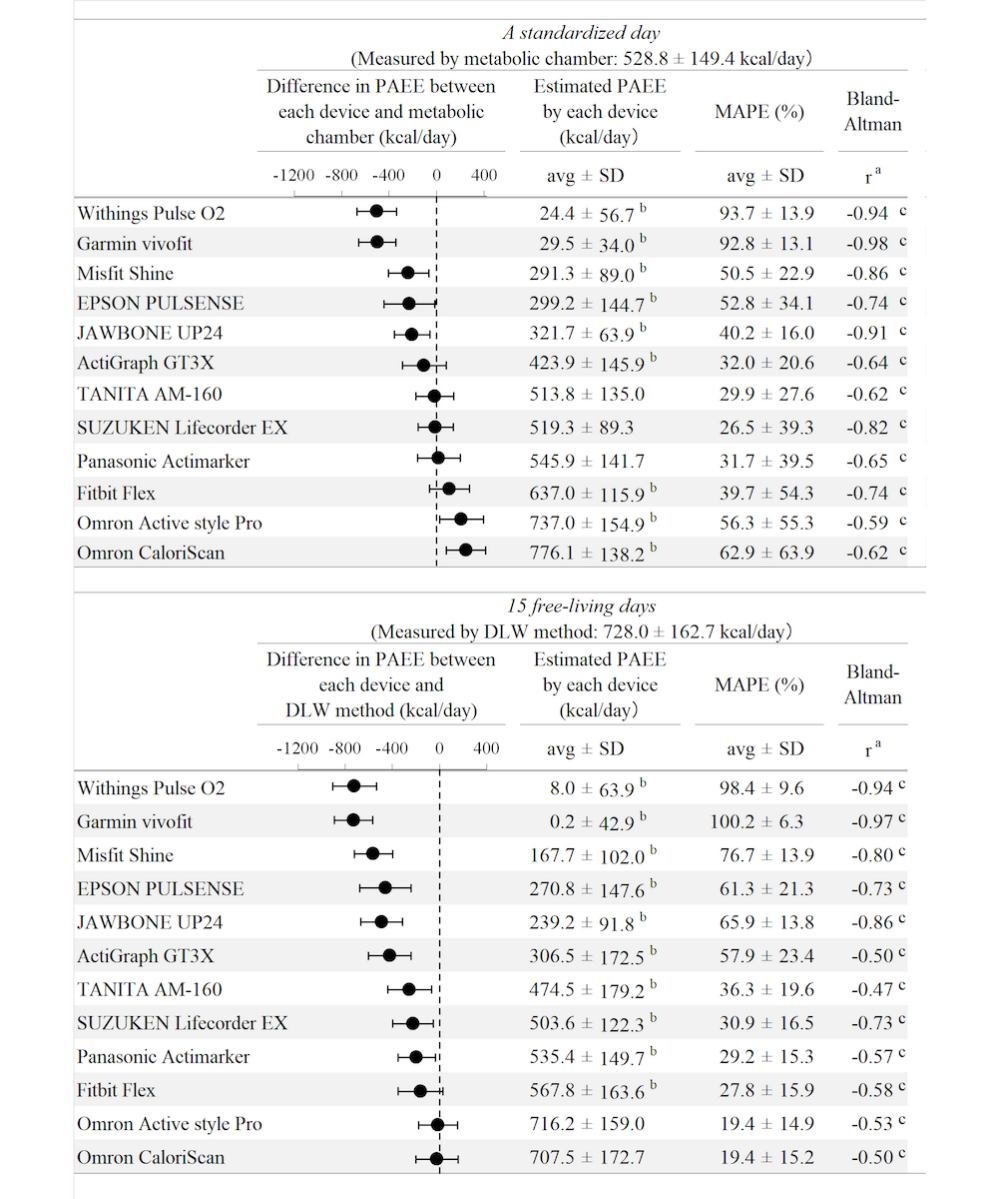
Differences between PAEEcha (physical activity energy expenditure) or PAEEdlw and each PAEEdev on a standardized day and 15 free-living days. Note: (a) The correlations between PAEEcha or PAEEdlw in x axis and delta as PAEEdev - PAEEcha or PAEEdev - PAEEdlw in y axis were showed. (b) *P*<.05 vs PAEEcha or PAEEdlw. (c) *P*<.05 in correlations. PAEE: physical activity energy expenditure; MAPE: mean absolute percentage error; avg: average; SD: standard deviation.

During the standardized day, the PAEE_cha_ was 528.8±149.4 kcal/day. All devices except the TANITA AM-160 (513.8±135.0 kcal/day; *P*>.05), SUZUKEN Lifecorder EX (519.3±89.3 kcal/day; *P*>.05), and Panasonic Actimarker (545.9±141.7 kcal/day; *P*>.05) showed significant differences in PAEE_dev_ compared with PAEE_cha_ (Figure 1). Moreover, 6 devices significantly underestimated values, whereas 3 devices significantly overestimated values. The Withings Pulse O2 (24.4±56.7 kcal/day) and Garmin Vivofit (29.5±34.0 kcal/day) showed large gaps in PAEE_cha_, with MAPEs of 93.7±13.9% and 92.8±13.1%, respectively. Moreover, all devices showed systematic errors with high negative correlation coefficients on the Bland-Altman plots (Figure 1).

**Figure 2:**
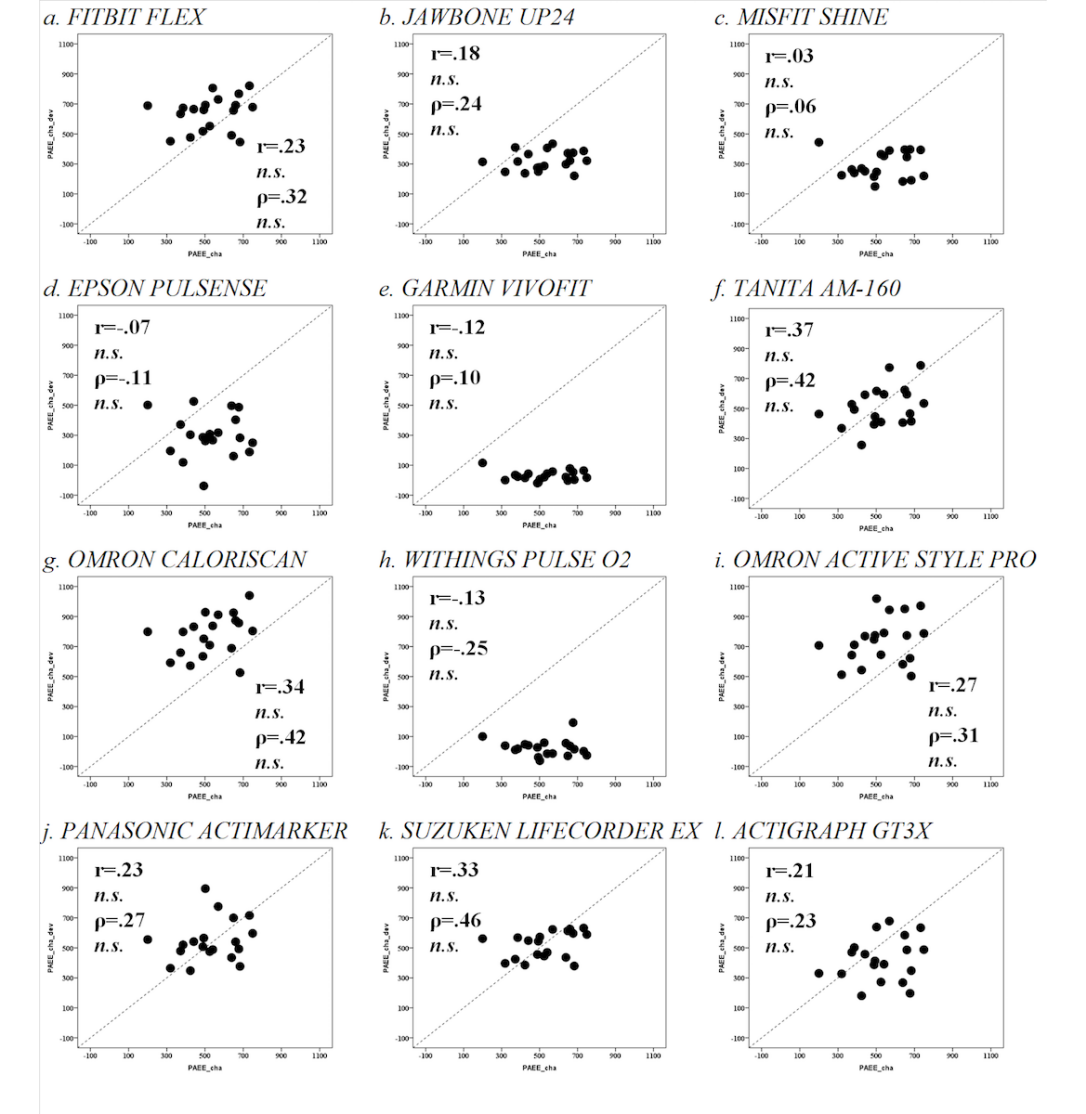
Correlation between PAEEcha (physical activity energy expenditure) and PAEEdev during 1 standardized day. Scattered plots between PAEEcha (x-axis) and PAEEdev (y-axis) during 1 standardized day. There was no significant correlation according to Pearson and Spearman tests. n.s.: nonsignificant.

No devices showed a significant correlation with PAEE_cha_ according to both Pearson and Spearman correlation tests (Figure 2). Regarding PAL, all devices except the TANITA AM-160 (1.51±0.07; *P*>.05), Panasonic Actimarker (1.56±0.08; *P*>.05), and SUZUKEN Lifecorder (1.55±0.04; *P*>.05) showed significant differences in PAL compared with PAL_cha_ (1.56±0.17; Tables 3 and 4). No devices showed a significant correlation with PAL_cha_ according to both Pearson and Spearman correlation tests (Tables 3 and 4). PAEE/body weight also showed similar results for PAL (Tables 3 and 4). Moreover, similar results were obtained in partial correlation test using body weight as a control variable.

**Table 3:** The comparison between PAL^a^_cha_ and PAL_dev_, PAEE^b^_cha_/wt (physical activity energy expenditure) and PAEE_dev_/wt.

Devices	A standardized day			
	PAL_cha_: 1.56 ± 0.17		PAEE_cha_/wt: 9.2 ± 2.4 kcal/kg/day	
	Value, average (SD)	Pearson correlation	Value, average (SD)	Pearson correlation
Withings Pulse O2	1.13 (0.04)^c^	0.08	0.5 (1.0)^c^	0.02
Garmin vivofit	1.13 (0.02)^c^	-0.27	0.5 (0.6)^c^	-0.37
Misfit Shine	1.30 (0.06)^c^	-0.19	5.1 (1.5)^c^	-0.25
EPSON PULSENSE	1.32 (0.11)^c^	0.08	5.4 (2.8)^c^	0.07
JAWBONE UP24	1.39 (0.06)^c^	-0.19	5.6 (1.0)^c^	-0.14
ActiGraph GT3X	1.47 (0.09)^c^	-0.29	7.2 (1.8)^c^	-0.26
TANITA AM-160	1.51 (0.07)	-0.13	8.8 (1.4)	-0.10
SUZUKEN Lifecorder EX	1.55 (0.04)	-0.30	9.0 (0.8)	-0.27
Panasonic Actimarker	1.56 (0.08)	-0.34	9.4 (1.5)	-0.26
Fitbit Flex	1.63 (0.06)^c^	-0.38	11.0 (1.2)^c^	-0.39
Omron Active style Pro	1.74 (0.07)^c^	-0.44	12.7 (1.4)^c^	-0.30
Omron CaloriScan	1.78 (0.07)^c^	-0.29	13.4 (1.2)^c^	-0.24

^a^PAL: physical activity level.

^b^PAEE: physical activity energy expenditure.

^c^*P*<.05 vs PAL_cha_ or PAEE_cha_/wt.

**Table 4:** The comparison between PAL^a^_dlw_ and PAL_dev_, PAEE^b^_dlw_/wt and PAEE_dev_/wt.

Devices	15 free-living days			
	PAL_dlw_: 1.73 ± 0.21		PAEE_dlw_/wt: 12.8 ± 3.1 kcal/kg/day	
	Value, average (SD)	Pearson correlation	Value, average (SD)	Pearson correlation
Withings Pulse O2	1.12 (0.04)^c^	-0.24	0.2 (1.1)^c^	-0.14
Garmin vivofit	1.11 (0.03)^c^	-0.08	0.0 (0.7)^c^	-0.07
Misfit Shine	1.22 (0.06)^c^	-0.02	2.9 (1.6)^c^	0.11
EPSON PULSENSE	1.30 (0.10)^c^	-0.31	4.7 (2.5)^c^	-0.05
JAWBONE UP24	1.31 (0.07)^c^	-0.30	4.1 (1.4)^c^	-0.10
ActiGraph GT3X	1.37 (0.13)^c^	0.11	5.2 (2.7)^c^	0.25
TANITA AM-160	1.48 (0.11)^c^	-0.01	8.1 (2.4)^c^	0.10
SUZUKEN Lifecorder EX	1.53 (0.08)^c^	-0.12	8.7 (1.5)^c^	0.07
Panasonic Actimarker	1.56 (0.10)^c^	0.26	9.3 (2.1)^c^	0.39
Fitbit Flex	1.57 (0.11)^c^	-0.08	9.8 (2.3)^c^	0.13
Omron Active style Pro	1.72 (0.10)	0.14	12.4 (1.9)	0.35
Omron CaloriScan	1.71 (0.09)	-0.07	12.2 (1.7)	0.11

^a^PAL: physical activity level.

^b^PAEE: physical activity energy expenditure.

^c^*P*<.05 vs PAL_dlw_ or PAEE_dlw_/wt.

### Doubly Labeled Water Study

**Figure 3:**
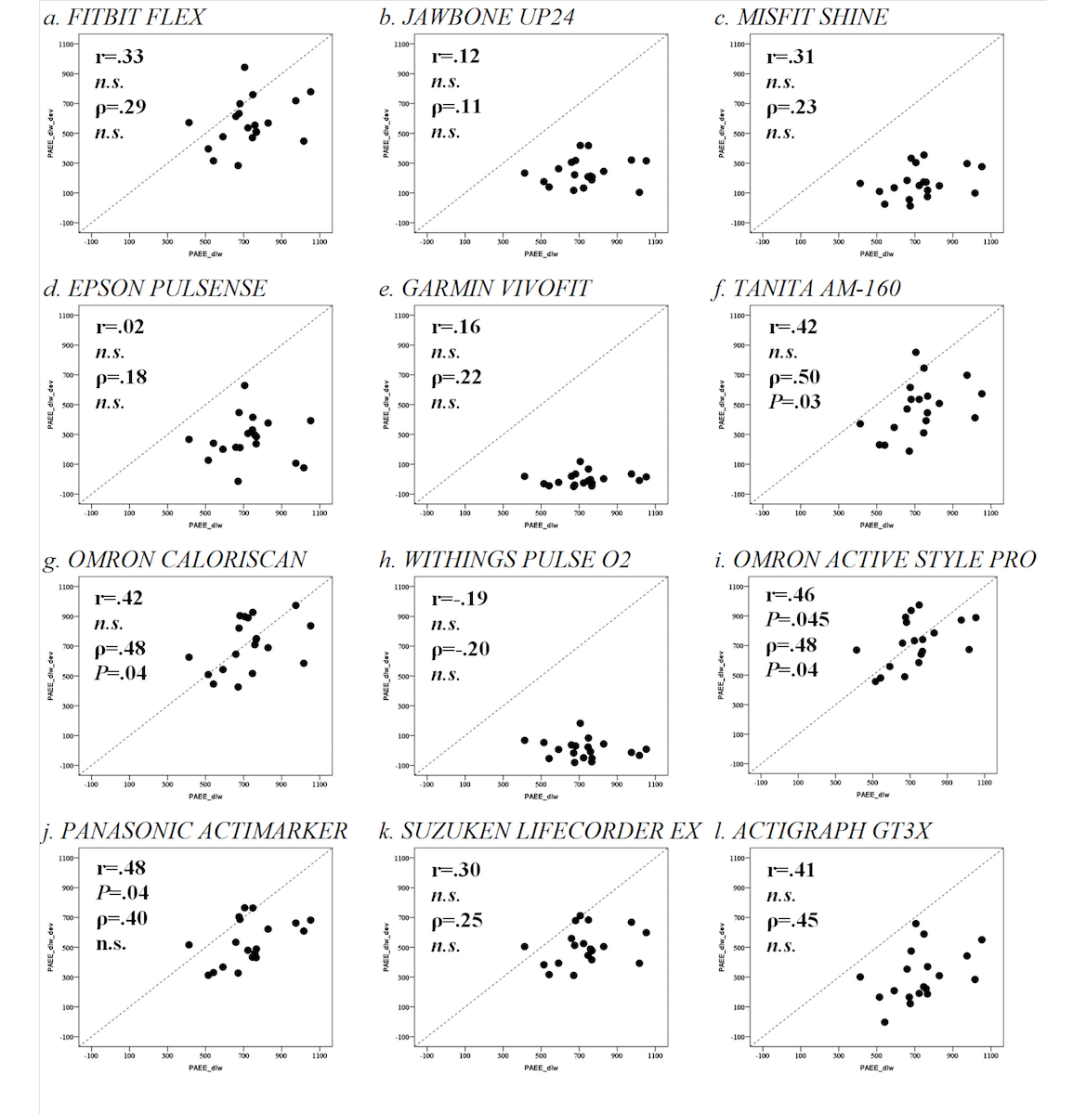
Correlation between PAEEdlw (physical activity energy expenditure) and PAEEdev during 15 free-living days. Scatter plots for PAEEdlw (x-axis) and PAEEdev (y-axis) during 15 free-living days. Upper and lower values for r and ρ resulting from Pearson and Spearman correlation tests, respectively, are shown. n.s.: nonsignificant.

During the 15 free-living days experiment, the PAEE_dlw_ was 728.0±162.7 kcal/day. The PAEEs from all devices except the Omron Active style Pro (716.2±159.0 kcal/day; *P*>.05) and Omron CaloriScan (707.5±172.7 kcal/day; *P*>.05) were significantly underestimated (Figure 1). Only 2 devices, the Omron Active style Pro (r=0.46; *P*=.045) and Panasonic Actimarker (r=0.48; *P*=.04), showed significant positive Pearson correlations. In addition, 3 devices, the TANITA AM-160 (ρ=.50; *P*=.03), Omron CaloriScan (ρ=.48; *P*=.04), and Omron Active style Pro (ρ=.48; *P*=.04), can be ranked in PAEE_dev_ (Figure 3). On the other hand, systematic biases indicated by Bland-Altman plots were observed for all devices with negative coefficients (Figure 1). Regarding PAL, all devices except the Omron Active style Pro (1.72±0.10; *P*>.05) and Omron CaloriScan (1.71±0.09; *P*>.05) showed significant differences in PAL_dev_ compared with PAL_dlw_ (1.73±0.21; Tables 3 and 4). No devices showed a significant correlation with PAL_dlw_ according to both Pearson and Spearman tests (Tables 3 and 4). PAEE/body weight also showed results similar to those of PAL (Tables 3 and 4). Moreover, similar results with partial correlation were obtained using body weight as a control variable.

**Table 5:** The comparison between PAEE^a^_cha_ and the unique PAEE parameters extracted from each consumer-based wearable device.

Number	Devices	Item	Standardized day (PAEE_cha_: 528.8 ± 149.4 kcal/day）	
			Value, average (SD)	Pearson correlation
1	Fitbit Flex	N/A^b^	—^c^	—
2	JAWBONE UP24	active energy expenditure	503.3 (77.9 )	0.30
3	Misfit Shine	N/A	—	—
4	EPSON PULSENSE	active energy expenditure	416.2 (173.1)	0.14
5	Garmin vivofit	exercise energy expenditure (web)	212.4 (44.4)	0.32
6	TANITA AM-160	active energy expenditure	726.8 (168.7)	0.39
7	Omron CaloriScan	active energy expenditure (web)	774.3 (137.1)	0.40
8	Withings Pulse O2	activity energy expenditure	318.4 (54.8)	0.23

^a^PAEE: physical activity energy expenditure.

^b^Fitbit and Misfit Shine were not available for unique PAEE parameters in their app and website.

^c^Not applicable.

### Unique PAEE by Consumer-Based Devices

On the standardized day, we also compared the PAEE_cha_ with the unique PAEE parameters obtained by 6 of the 8 consumer-based devices (Table 5). The absolute values from each device were not compared with PAEE_cha_ because we could not find any information about these parameters and could not define the value as PAEE. None of the parameters showed a significant correlation with PAEE_cha_.

## Discussion

### Principal Findings

We examined the validity of 12 consumer-based and research-grade wearable devices for estimating PAEE using a metabolic chamber and the DLW method as standard methods. On the standardized day, most of the wearable devices showed significant differences in PAEE when compared with PAEE_cha_ (MAPE 26.5%-93.7%). Moreover, all wearable devices except the Omron CaloriScan and Omron Active style Pro significantly underestimated values during 15 free-living days (MAPE 19.4%-100.2%). These results were similar, even for PAL. The number of wearable devices with significant differences in PAEE compared with the standard criteria in this study was greater than the number of devices with significant differences in TEE in our previous study using same 12 devices; we found that only 2 devices during the standardized day and 4 devices during 15 free-living days showed significant differences in TEE compared with the standard criteria [5]. These results showed that wearable devices had lesser accuracy when estimating PAEE than TEE, which included the BMR.

### Comparison With Previous Studies

Several studies have evaluated the validity of EE estimated by wearable devices during some activities [7-14]. Most of these studies were conducted during very short structured activities in laboratories. For the most studied device (Fitbit), there were many inconsistent results such as overestimated EE [4,33,34], underestimated EE [11,12], and comparable EE [8]. It has also been reported that the EE estimations based on the Fitbit were largely different depending on the activity types performed during those studies [8,12]. These discrepancies may have been dependent on the differences in the standard criteria, EE assessment method, and selected activities. In this study, the PAEE estimated by the Fitbit Flex was somewhat comparable with standard PAEEs during a standardized day and during 15 free-living days in consumer-based wearable devices, which was consistent with the results of the Fitbit Zip [11]. Furthermore, in this study, the JAWBONE UP24 underestimated PAEEs during both experiments, which was consistent with the results of previous studies [7,11]. However, the Misfit Shine and Garmin Vivofit underestimated PAEE during this study but overestimated PAEE during previous studies [7,9]. Attention is necessary when directly comparing the present results of this study with the previous results because what was used to evaluate PAEE was slightly different. We evaluated TEE−BMR−TEE×0.1 as PAEE (ie, net EE with PA); however, most previous studies that evaluated EE included the BMR or REE during experimental activities as PAEE. We also compared the unique indices of PAEE provided by several devices as PAEE_cha_ (Table 5). These were indicated on the app as active EE or exercise EE. However, no parameters were significantly correlated with PAEE_cha_. Most evidence that demonstrated the relationship between PA and risk reduction of disease based on epidemiological studies were described as the amount of PA but not as the TEE. Therefore, it is important to accurately assess daily PAEE in terms of preventive medicine and public health.

### Underestimation Under Free Living

In a comparison of the results of the standardized day and those of 15 free-living days, all wearable devices except the Omron CaloriScan and Omron Active style Pro underestimated PAEE for 15 free-living days, whereas 6 devices underestimated PAEE, and 3 devices overestimated PAEE on the standardized day. Because TEE measurements using the metabolic chamber have been reported as not significantly different from TEE measured by DLW methods on the same days [35], our results were not caused by different criteria for the TEE assessment. Underestimation by most devices during 15 free-living days may have been partly caused by the nonwearing time. We calculated the average PAEE during the nonwearing time (PAEE_nonwear_) by multiplying the nonwearing time by MET corresponding to the PA performed [20] based on the daily log recorded by participants. Even if PAEE_nonwear_ derived from each wearable device were added to each PAEE_dev_, PAEE would have remained underestimated. This means that many types of PA are underestimated during free-living days.

It has been reported that cycling and washing laundry are underestimated by wearable devices [8,12]. Moreover, standing that does not produce acceleration may be classified as sedentary behavior [36]. These types of PA during free-living days may have caused underestimation of PAEE in this study. Although early consumer-based wearable devices for estimating PA relied on movement sensors alone (eg, accelerometers), more recently developed wearable devices integrate several physiological or geographical outputs, including heart rate, skin temperature, galvanic skin response, and a global positioning system [37]. PAEE that cannot be captured by an accelerometer may be accurately estimated using these multisensor wearable devices in the future. Another reason for the underestimation of PAEE during free-living days could have been transition in postures (eg, sit-to-stand), transition in directions, and acceleration and deceleration during movements. Recent studies have suggested that significant additional EE is associated with changing directions and/or changing postures [38-41], and those transitions are often observed during free-living days [42,43]. However, those elements were not usually considered to establish and validate PA monitors. To assess actual PAEE during daily life, it is necessary to continuously evaluate the validity of these sensors for estimating PAEE.

### Perspectives

Wearable devices can be powerful tools that provide not only individual information but also large-scale population data on a global scale. Most wearable devices can connect to the internet through an app on a user’s mobile phone and collect data. Using 68 million days of step count data from 717,517 users of the Argus Smartphone app, Althoff et al [44] showed that inequality in PA within a country was associated with the prevalence of obesity in the population. Moreover, multiple aspects of health behavior need to be monitored simultaneously and continually because our health outcomes resulted from various health behaviors that included not only PA but also daily diet, smoking, and sleep [45]. Under such circumstances, it is important to be able to properly evaluate the multilateral health behavior and physiological parameters globally. However, some problems have been highlighted by the continuous wearing of such a device. One-third of owners of a consumer-based wearable device stopped using it within 6 months [15]. Therefore, it is necessary to enhance continuity and strive to maintain and improve health outcomes through various other approaches.

### Limitations

There were some limitations to this study. First, the sample size was small and restricted to normal-weight individuals; therefore, results cannot be generalized to obese or lean people. Comprehensive validation extending to other populations with various PALs is required. Because it was expected that some types of PA were underestimated and some were overestimated by wearable devices, different PA situations may lead to different results. Second, we could not examine the validity of all wearable devices for all types of activity during a standardized day. Different settings using different intensities and other types of activities may lead to different results. We also need to confirm the results in different settings or examine the validity of each activity performed during a standardized day to reveal the causes of the underestimation and overestimation. Third, BMR values estimated by several wearable devices were obtained as whole-day values with stable situation. This was not supposed by the manufacturers; therefore, we might have used BMR incorrectly for several devices, which might have led to erroneous estimations of PAEE because it was calculated by subtracting the BMR from the TEE. Therefore, comparisons of absolute values of PAEE for these devices in this study must be interpreted with caution.

### Conclusions

In conclusion, most wearable devices showed PAEEs that were significantly different from those estimated using gold standard methods during a standardized day and 15 free-living days. It is possible that the PAEE of some PA is underestimated during free-living situations by wearable devices. The development of wearable devices that can accurately estimate PAEE will lead people to use them as motivational tools. Moreover, this will allow researchers to precisely understand PA in an observational study or intervention study, thereby leading to public health recommendations based on scientific evidence.

## Abbreviations

AMED: Japan Agency for Medical Research and Development
BMI: body mass index
BMR: basal metabolic rate
DIT: dietary-induced thermogenesis
DLW: doubly labeled water
EE: energy expenditure
FQ: food quotient
IRMS: isotope ratio mass spectrometer
MAPEs: mean absolute percent errors
MET: metabolic equivalent
PA: physical activity
PAEE: physical activity energy expenditure
PAL: physical activity level
rCO _2_: carbon dioxide production rate
REE: resting energy expenditure
TBW: total body water
TEE: total energy expenditure
VCO _2_: carbon dioxide output
VO _2_: oxygen uptake
